# The direct cost of care among surgical inpatients at a tertiary hospital in south west Nigeria

**DOI:** 10.11604/pamj.2014.18.3.3177

**Published:** 2014-05-01

**Authors:** Olayinka Stephen Ilesanmi, Akinola Ayoola Fatiregun

**Affiliations:** 1 Department of Community Medicine, University College Hospital, Ibadan, Nigeria; 2 Department of Epidemiology and Medical Statistics, Faculty of Public Health, College of Medicine, University of Ibadan, Ibadan, Nigeria

**Keywords:** Cost, surgery, anaesthesia, health care, resource, hospital bill, hospital length of stay

## Abstract

**Introduction:**

This study was conducted to assess the direct cost of care and its determinants among surgical inpatients at university College Hospital, Ibadan.

**Methods:**

A retrospective review of records of 404 inpatients that had surgery from January to December, 2010 was conducted. Information was extracted on socio-demographic variables, investigations, drugs, length of stay (LOS) and cost of carewith a semi-structured pro-forma. Mean cost of care were compared using t-test and Analysis of variance (ANOVA). Linear regression analysis was used to identify determinants of cost of care. Level of significance of 5% was used. In year 2010 $1 was equivalent to 150 naira ($1=₦ 150).

**Results:**

The median age of patients was 30 years with inter-quartile range of 13-42 years. Males were 257(63. 6%). The mean overall cost of care was ₦66,983 ± ₦31,985. Cost of surgery is about 50% of total cost of care. Patient first seen at the Accident and Emergency had a significantly higher mean cost of care of ß = ₦17,207(95% CI: ₦4,003 to ₦30,410). Neuro Surgery (ß=₦36,210), and Orthopaedic Surgery versus General Surgery(ß=₦10,258),and Blood transfusion (ß=₦18,493) all contributed to cost of care significantly. Increase of one day in LOS significantly increased cost of care by ₦2,372. 57.

**Conclusion:**

The evidence evaluated here shows that costs and LOS are interrelated. Attempt at reducing LOS will reduce the costs of care of surgical inpatient.

## Introduction

Health care costs have been on the increase over the past few years in many developing countries. This expanding cost of care has been a source of concern to health care providers and managers. [1],This has made reduction in hospital length of stay (LOS) to assume great importance[2–6]. LOS has been designated as a universal metric for gauging this cost of care as prolonged hospitalization tends to increase aggregated health-care expenditures including the opportunity cost of hospital stay[2, 4]. The trend in most developed countries has therefore been to promote shortened hospital stay where it is possible [6].

Increase in length of hospitalization tends to increase aggregated health-care expenditures [2, 7]. A quarter of the respondents studied in a Nigerian population experienced financial hardship in settling their medical bills, invaluable assets of the respondents such as farm lands were sold in the quest of seeking health care [8]. Some surgical patients could not come for follow up due to financial constraint as a result of long hospitalization. Some patients were also detained in the hospital due to inability to pay their bills[9]. Difficulty has also been seen in carrying out post-operative investigations, purchase of post-operative drugs and payment of hospital bill after a long stay in the hospital [1].

Hospital stay can drain the patients financially [10]. Other than increased health care expenditure there is an increased risk of nosocomial infections [11], adverse drug reactions can occur particularly with intravenous drug use and complications related to other invasive procedures. These risks have been shown to increase with increasing duration of hospital stay[12, 13]. This will further increase health care expenditure. Higher ASA has been found to be associated with high cost of care[14]. Variables that describe patient treatment activities, administrative processes, testing procedures and general operational attributes involving various departments of a healthcare organization can provide decision makers with vital information regarding resource effectiveness in patient care [15].

The absence of the various health insurance schemes that are available in the developed countries has made health care financing burdensome in the developing countries[16]. Health care financing has been a problem in Sub – Saharan Africa due to increase demand for health services and rising health care cost and low coverage of the National Health Insurance Scheme [17]. This has made out-of pocket expenditure the common form of health care financing[16, 18]. Out-of pocket health expenses reduces healthcare uptake or utilization, and the cost-effectiveness of the healthcare system[9, 16]. The nature of health service received also has implication for health care expenditure [19, 20].

Unfortunately, the number of studies that report the cost of care of surgical inpatients is quite small, as few economic analyses relating to management of surgical inpatients have been conducted in Nigeria. Therefore, it is important to know the cost of care and identify its determinants. This will assist in identifying ways to reduce the financial burden. This study aimed to estimate the direct cost of hospital stay and to document the determinants of cost of care to surgical inpatients at university College Hospital, Ibadan.

## Methods

**Study design**: we carried out a retrospective review of records of patients who had surgery between January and December, 2010 at the University College Hospital, Ibadan.

**Study area**: the study was carried out at the University College Hospital (UCH), Ibadan. UCH is a tertiary health institution with eight hundred and fifty beds it serves as referral centre for other hospital within and outside Oyo State. The hospital has about 60 service and clinical Departments and runs 131 consultative out-patient clinics a week in 50 specialty and sub-specialty disciplines.

The Surgical sub specialities in the University College Hospital include General Surgery, Paediatric Surgery, Plastic Surgery, Urology, Orthopaedics and Neurosurgery. There are four major surgical wards in the hospital each accommodating minimum of 30 patients each. There are seven operating theatres in the Main Theatre suites, two in the Gynaecology suites and one in Accident and Emergency. An average of 300 surgeries is performed monthly out of which about 120 are electives under General Anaesthesia. There are over 165 hospital consultants and in-patient admissions exceeding 10,000 while out-patient clinic attendances approximates are over 170,000 a year [22].

**Inclusion criteria**: A total of 3,312 patients had surgery in the main theatre of University College Hospital, Ibadan from January to December 2010. In all, 1,571 primarily surgical patients who belong to the core surgical sub-specialities of General Surgery, Paediatric Surgery, Plastic Surgery, Urology, Orthopaedics and Neurosurgery, meet the inclusion criteria. In all only 420 case notes were available for review.

**Exclusion criteria**: Patients that were excluded include those that had day case surgery and patients admitted in the private suit. Others were Ear, Nose and Throat patients and Ophthalmology patients.

### Data collection

A structured pro-forma was used to extract information on socio-demographic variables, admission and surgical processes, peri-operative and post-operative conditions and duration of hospital stay. The hospital numbers of surgical inpatients seen from January to December, 2010 were obtained from the main operating theatre's surgery register. The register contained the hospital number, name, age, sex, operation done and surgeon, anaesthetist and type of anaesthesia. The hospital number collected was used to locate patient's case notes from the record unit of the Surgery outpatient clinic. LOS was the number of days from admission to discharge.

### Data management

Data was entered, cleaned and analysed with SPSS version 15. Frequencies and proportions were generated. Mean cost of care were compared using t-test and Analysis of variance (ANOVA). Linear regression analysis was used to identify determinants of cost of care. The dependent variable was the direct cost of hospital stay. The independent variables included: Socio-demographic variables like age, sex, marital status, religion, level of education, occupation, case specific variables e. g diagnosis type of surgery done, co morbidities, anaesthesiologists grading i. e. American Society of Anaesthesiologists (ASA) grading of patients physical status pre-operative and LOS.

### Cost of care estimation

The cost of patient`s registration, consultations, surgery, anaesthesia, investigations and other related bills were extracted from the hospital`s price list. Costs of laboratory investigations were estimated by collating all the available investigation result seen in the case note and the price were checked from the hospital`s price list. Cost of drugs were based on the hospital cost of each drugs prescribed before and after surgery. This was done by contacting the hospital pharmacy. Cost of consumables like syringes and needle were imputed from the number of times intravenous drugs were used. Costs of intravenous cannulars were based on the approximate number of times it was changed during admission. All the costs were calculated by using the hospital billing documents at the period studied. Daily charges of bed fee and feeding fee were calculated and multiplied by the total number of days spent on admission. The case notes were scrutinized for every duplicate copy of receipt or bank teller seen and any record of payment. Summation of all the cost was done to estimate the direct cost of patient care. Data was presented using frequency tables. Level of statistical significance was set at 5%. In year 2010 $1 was equivalent to ₦150.

**Ethical considerations**: Approval for the study was received from the University of Ibadan/University College Hospital ethical review board.

## Results

Out of the 3,312 patients registered to have had surgery in the main theatre of University College Hospital, Ibadan from January to December 2010, only 1,571 cases met the inclusion criteria. In all, 420 case notes were available and retrieved out of which 16 case notes contained incomplete information on key variables of interest. Only 404 case notes were analysed, while the remaining 1,167 were not.

### Comparison of mean cost of care across socio-demographic variables

The mean overall cost of care was ₦66,983 ± ₦31,985. Comparison of mean cost of care across socio-demographic variables is as shown in Table 1. The overall mean cost of care is higher for patients 40 years and above ₦74,233 ± ₦31,422 compare to patient less than 40 years ₦59,136 ± ₦30,788 (P

**Table 1:** Comparison of mean cost of care of surgical inpatient across socio-demographic variables

Variables	N	Percent	Mean± Standard Deviation ₦	Test statistic	p-value
**Age group**					
<40	194	48.0	59,136 ± 30,788	-4.872*	<0.001
≥40	210	52.0	74,233 ± 31,422		
**Sex**					
Male	257	63.6	68,723 ± 33,118	1.448*	0.149
Female	147	36.4	63,941 ± 29,766		
**Marital Status**					
Single	208	51.5	60,868 ± 30,949	8.468**	<0.001
Married	183	45.3	73,967 ± 31,986		
Widowed	13	3.2	66,517 ± 30,422		
**Religion**					
Christianity	261	64.6	64,834 ± 30,978	-1.830*	0.068
Islam	143	35.4	70,906 ± 33,501		
**Occupation**					
Retired Civil servants	16	4.0	76,713 ± 44,496	7.752**	<0.001
Others†	20	5.0	81,688 ± 35,243		
Infant/Child	43	10.6	43,526 ± 23,734		
Artisan	54	13.4	80,465 ± 28,101		
Civil Servant	61	15.1	65,524 ± 27,765		
Business/Trader	85	21.0	71,735 ± 30,740		
Student	125	30.9	63,112 ± 31,700		

* T-test

** F-test (ANOVA)

† Others were house wives, clergies, and farmers

### Comparison of mean cost of care across department/unit of patient entry to the hospital, and selected surgical characteristics

Table 2 shows the Comparison of mean cost of care across department/unit of patient entry to the hospital, duration of main symptoms before presentation and other surgical characteristics. Patients admitted via the Accident and Emergency had a mean cost of care of ₦ 83,216 ± ₦32,018 followed by patients first seen at the Medical Out Patient (MOP) ₦64,616 ± ₦22,522(P<0. 05). Patients that had emergency Surgery had a mean cost of care of ₦76,420 ± ₦31,414, compared with patient with elective surgery with a mean cost of ₦58,770 ± ₦30,227(P<0. 05). Neuro-surgery patients had the highest mean cost of care of ₦106,245 ± ₦31,355 (P<0. 05). The differences in the mean cost of care of patients operated on by consultant were not significantly different from patients who were operated on by resident doctors.

**Table 2:** Comparison of mean cost of care across department/unit of patient entry to UCH, and other surgical characteristics

Variables	N	Percent	Mean± Standard Deviation ₦	Test Statistic	p-value
**Department / Unit of patient entry to UCH**					
SOP	178	44.1	60,393 ± 30,327	11.227**	<0.001
Accident and Emergency	128	31.8	83,216 ± 32,018		
GOPD	35	8.7	56,040 ± 23,577		
OTCHEW	25	6.2	58,823 ± 35,905		
Others	22	5.4	54,265 ± 22,770		
MOP	16	4.0	64,616 ± 22,522		
**Surgery Type**					
Elective	216	53.5	58,770 ± 30,227	-5.748*	<0.001
Emergency	188	46.5	76,420 ± 31,414		
**Surgery Group**					
General Surgery	209	51.7	62,901 ± 29,650	28.771**	<0.001
Orthopaedics Surgery	56	13.9	77,540 ± 27,126		
Urological Surgery	41	10.1	46,575 ± 22,435		
Plastic Surgery	58	14.4	58,850 ± 25,882		
Neurological Surgery	40	9.9	106,245 ± 31,355		
**Rank of Surgeon**					
Consultant	199	49.3	68,820 ± 33,673	1.137*	0.256
Resident	205	50.7	65,201 ± 30,232		

* T-test

** F-test (ANOVA)

### Comparison of mean cost of care across selected characteristics of subjects/ surgeries performed

Comparison of mean cost of care across selected characteristics of subjects/ surgeries performed are seen below in Table 3. Increasing ASA, General Anaesthesia, longer duration of surgery blood transfusion and higher blood loss were associated with higher mean cost of care (P<0. 05).

**Table 3:** Comparison of mean cost of care across selected characteristics of subjects/ surgeries performed

Characteristics	N	Percent	Mean± Standard Deviation ₦	Test Statistics	p-value
**ASA**					
I	227	56.2	57,738 ± 28,916	17.679**	<0.001
II	99	24.5	74,902 ± 31,189		
III	68	16.8	82,876 ± 29,710		
IV	10	2.5	90,384 ± 47,832		
**Anaesthesia**					
General Anaesthesia	281	69.6	71,776 ± 33,071	18.268**	<0.001
Local Anaesthesia	74	18.3	47,595 ± 21,676		
Other types of Anaesthesia†	49	12.1	68,782 ± 27,884		
**Duration of Surgery in hours (n=91)**					
1hour and below	21	5.2	58,701 ± 24,683	3.842**	0.025
Above 1 hour and less than 3 hours	47	11.6	73,391 ± 32,264		
3 hours and above	23	5.7	84,027 ± 30,984		
**Blood transfusion**					
Yes	79	19.6	85,440 ± 31,279	5.598*	<0.001
No	325	80.4	62,498 ± 30,557		
**Estimated Blood Loss during surgery**					
Below 100 mls	196	48.5	59,470 ± 28,958	14.573**	<0.001
100 to below 500 mls	136	33.7	68,596 ± 32,903		
500and below 1000 mls	53	13.1	78,324 ± 28,808		
**Laboratory investigations**					
Yes	386	95.5	66,447 ± 31,834	-1.564*	0.119
No	18	4.5	78,488 ± 33,979		
**Radiological Investigations**					
Yes	158	39.1	74,634 ± 74,634	3.921*	<0.001
No	246	60.9	62,070 ± 62,070		

* T-test

** F-test (ANOVA)

† Other types of anaesthesia were spinal, regional block, and conscious sedation.

### Linear regression analysis for determinants of cost of care

Linear regression analysis for socio-demographic determinants of cost of care is as shown in Table 4. After adjsusting for other variables respondents who were civil servant had a significantly lesser mean cost of care of ₦15,382 compared to artisan (95% CI: -₦26,757. 26 to -₦4,007. 30). Infants/Children also had a significantly lower mean cost of care of ₦29,942. 67 compared to artisan (95% CI:- ₦44,166. 92 to -₦15,718).

**Table 4:** Linear regression analysis for socio-demographic determinants of cost of care

Variables	B	p-value	95% Confidence Interval for B	
	₦		Lower Bound	Upper Bound
Age ≥ 40 years	10,239.96	0.081	-1,280.902	21,760.816
Female	-4,350.67	0.189	-10,847.073	2,145.726
Married versus Single	-555.31	0.912	-10,453.359	9,342.740
Widowed versus single	-7,029.98	0.496	-27,314.245	13,254.281
Business /Trader versus Artisan	-8,511.33	0.118	-19,192.889	2,170.225
Civil servant versus Artisan	-15,382.28	0.008	-26,757.260	-4,007.298
Infant/Child versus Artisan	-29,942.67	<0.001	-44,166.915	-15,718.423
Retired Civil servants versus Artisan	-4,520.46	0.618	-22,320.646	13,279.723
Students versus Artisan	-9,974.85	0.102	-21,922.709	19,73.008
Others versus Artisan	641.45	0.937	-15,195.708	16,478.600

### Linear regression analysis for surgical characteristics that determine cost of care

As shown in Table 5, after adjusting for other surgical characteristics respondents that were first seen at the Accident and Emergency had a significantly higher mean cost of care of ₦17,207. (95% CI: ₦4,003 to ₦30,410). Patients who had Neurological Surgery had a significantly higher mean cost of care of₦ 36,210 compared to patient who General Surgery (95% CI: ₦26,592 to ₦45,828). Orthopaedic Surgery patients had a significantly higher mean cost of care of ₦10,259 compared to General surgery patient (95% CI: ₦1,962 to ₦18,555). There is a significant decrease in mean cost of care of ₦14,040 in patients with Urological Surgery compared with patients who had General Surgery (95% CI: ₦-23,2971 to ₦-4,783).

**Table 5:** Linear regression analysis for surgical characteristics that determined cost of care

Variables	B	p-value	95% Confidence Interval for B	
	₦		Lower Bound	Upper Bound
First seen at A and E versus others*	17,206.949	0.011	4,003.414	30,410.485
First seen at GOPD versus others	3,576.192	0.632	-11,107.542	18,259.925
First seen at MOP versus others	7,987.514	0.375	-9,701.697	25,676.725
First seen at OTCHEW versus others	-830.952	0.920	-17,169.979	15,508.074
First seen at SOP versus others	3,433.122	0.582	-8,808.742	15,674.986
Emergency	5,938.209	0.069	-468.761	12,345.180
Orthopaedic versus General Surgery	10,258.505	0.016	1,962.251	18,554.759
Urology versus General Surgery	-14,039.848	0.003	-23,296.501	-4,783.194
Plastic versus General Surgery	-5,720.576	0.165	-13,797.391	2,356.239
Neurological versus General Surgery	36,210.437	<0.001	26,592.742	45,828.131

* Others were Children Outpatient Clinic, Gynaecology Clinic and Eye Clinic

Table 6 shows other surgical characteristics determining cost of care. Increase in ASA led to increase cost of care. There is a significant increase of ₦17,214 in the mean cost of care of patient that had General Anaesthesia compared with Local Anaesthesia (95% CI: ₦9,980 to ₦24,447). Patients with spinal, regional block and conscious sedation had a significant higher mean cost of care of 16,301 compared to patients with Local Anaesthesia (95% CI: ₦6,284 to ₦26,318). Duration of Surgery, blood transfusion, higher blood loss and radiological investigation increased mean cost of care significantly.

**Table 6:** Linear regression analysis for other surgical characteristics that determine cost of care

Variables	B	p-value	95% Confidence Interval for B	
	₦		Lower Bound	Upper Bound
ASAII versus ASAI	11,641.705	0.001	5,008.024	18,275.386
ASAIII versus ASAI	20,251.590	0.000	12,546.458	27,956.722
ASAIV versus ASAI	24,798.431	0.006	7,094.319	42,502.543
General Anaesthesia Versus Local	17,214.091	<0.001	9,980.708	24,447.474
Other types of anaesthesia Versus Local†	16,301.845	0.001	6,284.819	26,318.871
Duration of Surgery of above one hour to 3 hours versus one hour and less	4,280.342	0.332	-4,381.405	12,942.090
Duration of Surgery of three hours and above versus one hour and less	14,355.189	0.017	2,595.443	26,114.935
Blood transfusion versus no Blood transfusion	18,493.068	<0.001	10,846.978	26,139.158
Estimated Blood loss of 100-499 mls versus below 100 mls	3,232.841	0.463	-5,411.935	11,877.616
Estimated Blood loss of 500-999 mls versus below 100 mls	15,933.224	0.025	2,006.792	29,859.655
Presence of Laboratory investigation versus none	-2,033.686	0.769	-15,625.047	11,557.675
Presence of Radiological investigation versus none	10,213.737	0.001	4,393.033	16,034.441

† Other types of anaesthesia were spinal, regional block, and conscious sedation.

### Linear regression analysis for days between first visit and admission, days spent from admission to surgery and LOS as a determinant of cost of care

As shown in Table 7, increase of one day in LOS significantly increased cost of care by ₦ 2,372. 57. Days spent between first visit and admission and days spent from admission to surgerysignificantly reduced cost of care by ₦15. 32 and ₦936. 52 respectively.

**Table 7:** Linear regression analysis for days between first visit and admission, days spent from admission to surgery and LOS as a determinant of cost of care

Duration	B	p-value	95% Confidence Interval for B	
	₦		Lower Bound	Upper Bound
Days between first visit and admission	-15.32	0.030	-29.165	-1.467
Days spent from admission to surgery	-936.52	<0.001	-1323.898	-549.144
LOS(from admission to discharge)	2,372.57	<0.001	2120.219	2,624.918

## Discussion

Surgical inpatients are often faced with the question of the exact amount of money needed for their care. The hospital has existing document for exact cost of surgery. However, total cost of care cannot be easily arrived at since length of stay, investigations required and other determinants of cost of care are not known. This study has shown that cost of surgery is about 50% of the total cost of care. This information will help in advising the patients appropriately on the total cost of their care.

After adjusting for other socio-demographic variables civil servants and infant/children had significantly lesser mean cost of care compared to artisan. In this study, age 40 years and above had higher mean cost of care compared to those aged less than 40years. However, it is not statistically significant. Aging has been shown to be a significant predictor of health care cost due to increasing cardiovascular diseases[22].

Patients that were first seen at the Accident and Emergency had a significantly higher mean cost of care compared to others that were first seen at Children Outpatient Clinic, Gynaecology Clinic and Eye Clinic. Patients who had Neurological Surgery had a significantly higher mean cost of care compared to patient who had General Surgery. Urological Surgery also reduced cost of care significantly. Patient with ASA II, ASA III and ASA IV incurred higher cost of care compared to patient with ASA I. This is similar to findings of Daabiss that higher ASA is associated with higher cost of care[14]. General Anaesthesia and other types of anaesthesia like spinal, regional block and conscious sedation had more cost compared to patients that had Local Anaesthesia.

Patients with highest blood loss versus lowest blood loss and those who had blood transfusion as against those without blood transfusion incurred higher mean cost of care. This is similar to a previous study and it has led to seeking of alternatives to blood transfusion in the surgical setting to reduce the rising costs of care[23]. Request for Radiological investigations significantly increase cost of care. Similar to other studies in both developing and developed countries increasing LOS is also a significant predictor of cost of care[1–5, 24]

### Limitations

Due to the retrospective nature of this study missing case notes were encountered. However, comparison of missing case notes which were un-analysed and the analysed cases shows no significant difference in the age and sex of patients in the two categories. Hence, the outcome of the study can be generalized to surgical inpatients. Another limitation was that the study did not attach monetary value to indirect cost caused by surgery due to inability to work during hospitalization. We did not study this aspect, since the occupation recorded in the case note was done at the time of registration. Occupation might have changed at the time of the surgery. No objective tool exists for valuing time loss in the study population.

## Conclusion

This study has shown that cost of surgery is about 50% of the total cost of care. The factors determining increase mean cost of care were being civil servants, infant/Children, being first seen at the accident and emergency. ASA II and above and General Anaesthesia were also significant determinant of cost of care. Neurological surgery, blood loss above 500mls, blood transfusion and longer length of stay led to increase cost of care in this study. The evidence evaluated here suggests that costs and LOS are interrelated. Attempt at reducing LOS will reduce the costs of care as well. Prevention of blood transfusion will reduce the cost of care.
